# Gray matter volume and burnout severity among medical professionals

**DOI:** 10.1192/j.eurpsy.2022.1593

**Published:** 2022-09-01

**Authors:** J. Fujino, S. Tei, K. Abe, H. Takahashi

**Affiliations:** 1 Tokyo Medical and Dental University, Department Of Psychiatry And Behavioral Sciences, Tokyo, Japan; 2 Kyoto University, Department Of Psychiatry, kyoto, Japan

**Keywords:** burnout, medical professionals

## Abstract

**Introduction:**

Occupational burnout has become a pervasive problem in human services. Medical professionals are particularly vulnerable to burnout, which may lead to reduced motivation, medical errors, and voluntary absenteeism. To ensure effect functioning of medical systems, better understanding of burnout among medical professionals is warranted.

**Objectives:**

We aimed to investigate the structural brain correlates of burnout severity among medical professionals.

**Methods:**

Nurses in active service underwent structural magnetic resonance imaging. We assessed their burnout severity using self-reported psychological questionnaires. This study was approved by the Committee on Medical Ethics of Kyoto University and was conducted in accordance with the Code of Ethics of the World Medical Association.

**Results:**

The results reflected considerable individual differences in burnout severity in our sample. Our findngs revealed that the levels of burnout severity were associated with the regional gray matter volumes in brain areas such as ventromedial prefrontal cortex and insula.

**Conclusions:**

Since the outbreak of the COVID-19 pandemic, medical professionals have faced even greater stress. We hope that our findings will contribute to a better understanding of the mechanisms of burnout and offer useful insights for developing effective interventions to manage stress and burnout.

**Disclosure:**

No significant relationships.

